# Advanced Intelligent Control in Robots

**DOI:** 10.3390/s23125699

**Published:** 2023-06-19

**Authors:** Luige Vladareanu, Hongnian Yu, Hongbo Wang, Yongfei Feng

**Affiliations:** 1 Institute of Solid Mechanics of the Romanian Academy, 010141 Bucharest, Romania; 2 The School of Computing, Engineering and the Built Environment, Edinburgh Napier University, Edinburgh EH11 4BN, UK; h.yu@napier.ac.uk; 3 Academy for Engineering and Technology, Fudan University, Shanghai 200433, China; wanghongbo@fudan.edu.cn; 4 Faculty of Mechanical Engineering & Mechanics, Ningbo University, Ningbo 315211, China; fengyongfei@nbu.edu.cn

## 1. Introduction

Advanced intelligent control (AIC) is a rapidly evolving and complex field that poses significant challenges. It is a practically important field and has potential applications. In this context, this Special Issue aims to foster advancements in science and technology by addressing the theoretical and practical aspects of intelligent control techniques and their applications using advanced intelligent control in robots. The main purpose of this Special Issue is to gather in-depth research and explore new trends in the design, control and applications of the real-time control of intelligent sensor systems. These trends include the use of advanced intelligent control methods and techniques, as well as the integration of innovative multi-sensor fusion techniques into robots. These advancements are combined with various technologies such as computer vision; virtual and augmented reality (VR&AR); and intelligent communication including remote control, adaptive sensor networks, human–robot (H2R) interaction systems and machine-to-machine (M2M) interfaces.

This Special Issue highlights intelligent decision support systems (IDSS), including remote sensing and its integration with DSS, GA-based DSS, fuzzy set DSS, rough set-based DSS, intelligent-agent-assisted DSS, process mining integration in decision support, adaptive DSS; computer-vision-based DSS and sensory and robotic DSS in AIC in robots. This Special Issue is an extension of the previously published successful Special Issue entitled “Advanced Intelligent Control” and the book entitled “AIC through VIPRO Platforms”.

Special attention is paid to the utilization of new and emerging technologies with AIC that apply complex robotic systems, such as enhanced IoT technologies and applications in the 5G densification era; bio-inspired techniques for future manufacturing enterprise control; a cyber-physical systems approach to the cognitive enterprise; the development of the IT Industry 4.0 concept; industrial systems in the digital age; cloud computing; robotics; and automation. This Special Issue addresses applications such as human aid mechatronics, military applications, rescue robots, firefighting robots, rehabilitation robots, robot-assisted surgery, and domestic robots.

## 2. Review of the Contributions in This Special Issue

AIC in robots is an interdisciplinary field which combines and extends theories and methods from control theory, computer science, and operations research areas with the aim of developing controllers which are highly adaptable to significant unanticipated changes. In line with this goal, in this Special Issue, 21 papers have been carefully selected through a rigorous review process.

The first paper, entitled “Nonlinear Intelligent Control of Two Link Robot Arm by Considering Human Voluntary Components” [1], investigates a nonlinear intelligent control system of a two-link robot arm by considering the human voluntary components and the feed-forward characteristics of a human multi-joint arm. The proposed feedback controller uses the multi-joint viscoelasticity of the human arm, while the feed-forward controller is based on a support vector machine (SVR), and the stabilization controller is based on operator theory. The viscoelastic properties of the multi-joint arm are measured and analyzed through experiments. To reduce the influence and uncertainty caused by interference inside the controlled object, the control system is designed based on the operator theory. The experimental results of using a feed-forward controller based on a mechanical model are compared with those using a feed-forward controller based on an SVR.

“Rethinking Sampled-Data Control for Unmanned Aircraft Systems” [2] explores the recent advancements and challenges at the intersection of real-time computing and control and develops innovative reconsidered sampling strategies that can improve performance and resource utilization. The proposed design framework can efficiently integrate the computational and physical characteristics of the system, increase robust performance and avoid the pitfalls of event-triggered sampling strategies. The paper focuses on comparing the control performance of a multicopter Unmanned Aircraft System (UAS) using different sampling strategies varying in terms of the “co-design” of computing resources (sampling rate) and the holistic system performance. The unique benefits of the proposed co-regulation strategy on control performance, computational efficiency and system robustness in comparison to the traditional fixed-periodic, event-triggered and self-triggered controllers are highlighted. A co-regulation strategy is implemented to provide insight into how to design co-regulated systems for control engineers. The pitfalls of event-triggered and self-triggered sampling strategies on UASs are discussed. Quantitative evaluations of all of these strategies are conducted based on evaluation metrics that could reflect both control performance and computing costs.

“Indirect-Neural-Approximation-Based Fault-Tolerant Integrated Attitude and Position Control of Spacecraft Proximity Operations” [3] investigates Fault-Tolerant Integrated Attitude and Position Control of Spacecraft Proximity Operations in the presence of unknown parameters, disturbances and actuator faults. The authors propose a controller which combines a relative attitude control law and a relative position control law, which are designed by adopting neural networks (NNs) to approximate the upper bound of the lumped unknowns. The indirect neural approximation is used to approximate the upper bound of the lumped unknowns. A simulation study on a 6-DOF spacecraft is conducted, and the results indicate that the proposed neural adaptive fault-tolerant controller can achieve superior performance and good uncertainty rejection capability, which guarantees the successful implementation of the spacecraft proximity operation.

“Intelligent Tracking of Mechanically Thrown Objects by Industrial Catching Robot for Automated In-Plant Logistics 4.0” [4] aims to accelerate the transportation process and increase productivity through the optimized utilization of in-plant facilities. The authors develop a 3D simulated environment which enables users to throw objects with any mass, diameter or surface air friction properties in a controlled internal logistics environment. To observe trajectories more accurately, they create an enormous dataset of thrown object trajectories to train an encoder–decoder bidirectional Long Short-Term Memory network (LSTM) deep NN using multi-view geometry among simulated cameras. This research contributes an enhanced intelligent tracking algorithm that can predict the remaining 3D interception positions of a thrown object by observing its initial flight trajectory. To demonstrate the proposed method, the training and testing results obtained via the encoder–decoder bidirectional LSTM deep NN, trained through 1000/3000 throws with 50/100/300 epochs and 100/200 neurons, are analyzed.

“Control Design for Uncertain Higher-Order Networked Nonlinear Systems via an Arbitrary Order Finite-Time Sliding Mode Control Law” [5] proposes a novel Sliding Mode Control Law by considering uncertainties including parametric variations and matched bounded disturbances. The topology of the system network of one leader and four followers sharing information under the action of the distributed control protocols is illustrated. The consensuses in the positions, velocities and accelerations among the followers and leader are displayed, with the corresponding convergences of position errors, velocities error and acceleration errors. The simulation results confirm that the newly designed law is an interesting candidate for higher-order uncertain systems.

“The Hybrid Position/Force Walking Robot Control Using Extenics Theory and Neutrosophic Logic Decision” [6] investigates Hybrid Position/Force Walking Robot Control by applying the method to a hexapod walking robot. The authors apply the Extenics theory and Extension set to obtain clear separation of the properties and specific characteristics of the control methods required by the hexapod robot. The result is then used with Neutrosophic logic and DSmT (Dezert Smarandache Theory) to create a decision algorithm between kinematic and dynamic regulators for each leg of the hexapod robot during its walking phases. A control probability graph and equations are used to create the decision algorithm. A Matlab Simulink simulation study is conducted to demonstrate the proposed hybrid control algorithm.

“Model Predictive Control of a Novel Wheeled–Legged Planetary Rover for Trajectory Tracking” [7] develops an innovative Wheeled–Legged Planetary Rover for Trajectory Tracking, and a hybrid serial–parallel topology is utilized to realize a rigid–flexible coupling mechanism. The control strategy for the wheeled–legged rover includes a trajectory tracking module based on model predictive control, the steering strategy and the wheel speed allocation algorithm. A cosimulation model is established in both NX/Motion and Simulink software to verify the control strategy.

Smart Vehicle Path Planning Based on Modified PRM Algorithm [8] proposes a pseudo-random sampling strategy with the main spatial axis as the reference axis, optimizing the generation of sampling points, removing redundant sampling points, setting the distance threshold between road points, adopting a two-way incremental method for collision detections and optimizing the number of collision detection calls to improve the construction efficiency of the roadmap. The proposed PRM is verified and analyzed using a ROS-based test platform. Compared with the basic PRM algorithm, the modified PRM algorithm has advantages in terms of the speed with which the roadmap is constructed, path planning and path length.

“Human–Robot Cooperative Strength Training Based on Robust Admittance Control Strategy” [9] designs a stiffness adjusting law of the admittance model based on the biomechanics of knee joints. The designed control law can guide the user to use force correctly and reduce the stress on the joint soft tissue. It not only avoids excessive compressive force on the joint soft tissue, but also enhances the stimulation of quadriceps femoris muscles. A novel sitting and lying lower limb rehabilitation robot (LLR-II) is developed. To verify the function, feasibility and effectiveness of the proposed lower limb flexion and extension strength training, eight stroke survivors were selected to participate in the test experiment using the LLR-II robot. The experiment results show that the designed controller can effectively reduce the possibility of joint soft tissue injury and enhance the stimulation of the quadriceps, and this active training method is effective for exercising the quadriceps.

“sEMG-Based Gain-Tuned Compliance Control for the Lower Limb Rehabilitation Robot during Passive Training” [10] develops a surface-electromyography-based gain-tuned compliance control (EGCC) strategy for a lower limb rehabilitation robot based on the mapping function relationship between the normalized surface electromyography (sEMG) signal and the gain parameter. The experimental results demonstrate that the adoption of the EGCC strategy could significantly enhance the compliance of the robot end-effector by detecting the sEMG signal and improving the safety of the robot in different training modes. This indicates that the EGCC strategy has good application prospects in the rehabilitation robot field.

“Research on Monocular-Vision-Based Finger-Joint-Angle-Measurement” [11] considers an industrial monocular-vision-based knuckle-joint-activity-measurement system, with a short measurement time and the simultaneous measurement of multiple joints, applied to an existing computer-vision detection system. An Experimental Platform is designed to acquire high-quality multi-angle light-source-irradiated multivariate images. Through the PC image-processing algorithm, the images can be processed to segment finger-joint identifiers for the subsequent calculation of the finger-joint angle and length. Nine healthy male volunteers were recruited for the experiment, and three different finger-joint angles were detected using TS–HOMLDM to verify the monocular-vision-based finger-joint-angle measurement system. The experimental results show that the average angular deviation in the flexion/extension of the knuckle is a minimum of 0.43° and a maximum of 0.59°, and the average angular deviation in the adduction/abduction of the knuckle is a minimum of 0.30° and a maximum of 0.81°, which are all less than 1°.

“Navigation Path Based Universal Mobile Manipulator Integrated Controller” [12] proposes a versatile integrated controller which is able to execute motion planning in a stable manner in various environments with simultaneous control, leading to great benefits with regard to the execution time compared to the traditional sequential control method. To validate the proposed method, an experiment for motion planning towards the given target coordinates using the mobile manipulator robot in a simulation environment is conducted.

“Prediction of Metal Additively Manufactured Surface Roughness Using Deep Neural Network” [13] considers robotized product manufacturing technology by introducing 3D printing into the manufacturing process based on a prediction of Metal Additively Manufactured Surface Roughness using a deep neural network (DNN). It proposes a methodology to improve the quality of AM products based on data analysis through various analysis methods such as data pre-processing and DNN combined with sensor data used to predict surface roughness in the proposed methodology. The usefulness and feasibility of the proposed methodology are proved by the experimental data collected from the gas metal arc welding (GMAW)-WAAM system applied to a robotized product manufacturing technology.

“Method of Changing Running Direction of Cheetah-Inspired Quadruped Robot” [14] establishes a dynamic model of a quadruped robot and a two-level stability index system, including a minimum index system and a range index system. A two-level stability index system, including a minimum index and range index, is developed based on the dynamic model of the robot, and the optimization variables, including leg landing points, trunk movement trajectory and posture change rule, are determined.

“A Self-Collision Detection Algorithm of a Dual-Manipulator System Based on GJK and Deep Learning” [15] introduces AI technology into a control system based on the Gilbert–Johnson–Keerthi (GJK) algorithm. A dataset and trained deep neural network (DLNet) are generated to improve the detection efficiency. By combining DLNet and the GJK algorithm, a two-level self-collision detection algorithm (DLGJK) is developed to solve real-time self-collision detection problems in a dual-manipulator system with fast-continuous and high-precision properties. The experimental results show that compared to that with the global use of the GJK, the DLGJK significantly increases the detection efficiency in both single detection and working-path detection.

“Spherical Wrist Manipulator Local Planner for Redundant Tasks in Collaborative Environments” [16] proposes a path planner for manipulators to execute tasks with a redundant number of joints executing redundant tasks in workspaces shared with dynamic obstacles such as humans. An intuitive parameterization of the end-effector (EE) angular motion, which decouples the rotation of the third joint of the wrist from the rest of the angular motions, is presented. The path planner is developed by considering that the rotation of the third wrist joint must be decoupled from the rest of the EE angular motion, the resulting EE manipulator dynamics should behave as a linear dynamical system, the collision avoidance strategy must consider the entire surface of the manipulator and all the local planner parameters must have a physical meaning. The approach enables industrial and medical applications, in which robot stiffness and dexterity can greatly improve task efficiency.

“Detecting Machining Defects inside Engine Piston Chamber with Computer Vision and Machine Learning” [17] develops robotic industrial applications for automotive manufacturing with the main goal of replacing the visual inspection performed by a human operator with a computer vision application. A machine leaning algorithm which has conventional processing and a prediction method that uses a machine learning model is established. The results demonstrate that the robustness of image processing applications from the field of manufacturing can be considerably improved by replacing the classic method with the machine learning algorithm, which ensures greater flexibility in developing the backbone of the application, mainly consisting of PLC communication, socket services and a human–machine interface.

“Synchronous Control of a Group of Flying Robots Following a Leader UAV in an Unfamiliar Environment” [18] investigates a quadrotor drone group which follows an automatically flying leader with drones equipped with low-end cameras. This provides a considerable number of resources necessary to help people trapped in dangerous environments without risking the health or lives of rescuers. The main innovation is the structure of the multi-agent group of UAVs, with inexpensive followers without data exchange between actors and computational power requirements. The obtained results suggest that the organization of such tasks in an automatic system is realistic and, most importantly, effective.

“Improvement of Hexacopter UAVs Attitude Parameters Employing Control and Decision Support Systems” [19] conducts tests on Hexacopter Unmanned Aerial Vehicles to verify their operational parameters, hover flight, drone stability and reliability, including the aerodynamics and robustness at different wind speeds. The flight parameters extracted from the sensor systems, comprising accelerometers, gyroscopes, magnetometers, barometers, GPS antenna and EO/IR cameras, are analyzed. An innovative hexacopter platform architecture in two variants, equipped with avionic components and sensors, is developed. The results of the tests carried out both in the laboratory and in situ during the start–stop maneuvers of the hexacopter engines are described and discussed.

“A Hybrid Stacked CNN and Residual Feedback GMDH-LSTM Deep Learning Model for Stroke Prediction Applied on Mobile AI Smart Hospital Platform” [20] develops a stroke prediction model by combining AI techniques with the existing Internet of Medical Things (IoMT) on a Mobile AI Smart Hospital Platform to improve the quality of medical care that patients receive remotely at home. A mobile AI engine that implements AI-based cloud computing complexities, especially in real-time environments of AI technologies, is presented. A Hybrid LSTM with a Dense-Layer Deep Learning Model for Stroke Prediction is proposed. The algorithm is lightweight for the proposed mobile AI engine and facilitates continuous diagnostics and accurate GMDH–LSTM-based EEG signal prediction for IoMT-simulated inputs. The innovative AI mHealth app achieves high accuracy determined by a stacked CNN which reaches 98% for stroke diagnosis. The GMDH neural network proves to be a good technique for monitoring EMG signals, with an average accuracy of 98.60% and an average of 96.68% for signal prediction, and by extending the GMDH model and a hybrid LSTM with a dense-layer deep learning model, the accuracy can reach an average of 99%.

“Implementation of a Real-Time Object Pick-and-Place System Based on a Changing Strategy for Rapidly-Exploring Random Tree” [21] implements a six-degree-of-freedom (DOF) robot with an external camera and a two-finger gripper through an ROS-based real-time Pick-and-Place System and an improved Rapidly Exploring Random Tree (RRT) algorithm, named the Changing Strategy RRT (CS-RRT) algorithm. By implementing the proposed CS-RRT algorithm in the Open Motion Planning Library and according to the imported URDF file, MoveIt can perform motion planning for different robot manipulators; thus, the proposed method can be easily applied to other six-degree-of-freedom (DOF) robots using the ROS-based real-time Pick-and-Place System.
